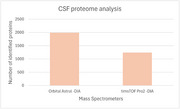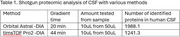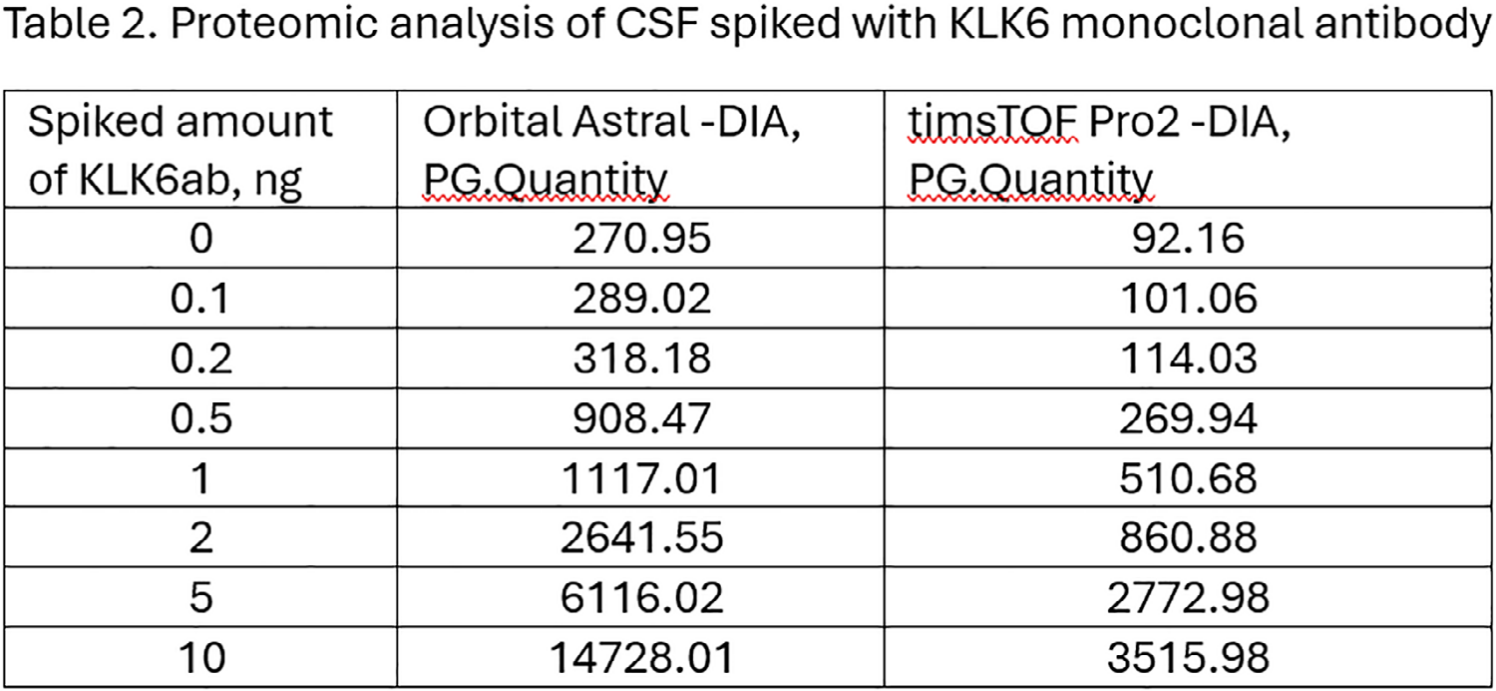# A mass spectrometric assay for detecting autoantibodies in cerebrospinal fluid with Data Independent Acquisition (DIA) utilizing new‐generation mass spectrometry

**DOI:** 10.1002/alz70856_096828

**Published:** 2025-12-24

**Authors:** Miyo K Chatanaka, Cassandra J. Wong, Antoninus Soosaipillai, Eleftherios P Diamandis

**Affiliations:** ^1^ University of Toronto, Toronto, ON, Canada; ^2^ Lunenfeld‐Tanenbaum Research Institute, Toronto, ON, Canada; ^3^ Sinai Health System, Toronto, ON, Canada

## Abstract

**Background:**

A hypothesis that has gained traction in Alzheimer's disease (AD) research is the autoimmune hypothesis, which posits that autoantibodies generated in the central nervous system attack neurons, leading to AD manifestations. To uncover such autoantibodies against neuronal proteins in AD, reliable, sensitive and specific analytical methods need to be developed.

**Method:**

Our method for the unbiased autoantibody identification in cerebrospinal fluid (CSF) includes the following steps: (1) capture of G‐class immunoglobulins from 10uL of CSF using protein G magnetic beads, (2) exposure to a brain tissue extract, leading to cognate antigen capture, (3) reduction by dithiothreitol and digestion with trypsin, (4) tryptic peptides extraction and subjection to analysis by mass spectrometry (MS) to identify the bound proteins. For method optimization, we used CSF spiked with known amounts of a monoclonal antibody against Kallikrein 6 (KLK6ab), which is abundant in normal CSF. MS was performed with two machines coupled to liquid chromatography: (a) an Orbitrap Astral MS operating in data‐independent acquisition (DIA) mode and 20min gradient, (b) a Bruker timsTOF Pro 2, operating in DIA mode and at either 22min or 44min gradient. Interpretation was done with Spectronaut.

**Result:**

We resolved the CSF proteome (20uL) with a shotgun approach and identified 1988.1 proteins with machine (a) in DIA (1/5 sample) and 1241.3 with (b) in DIA. We resolved the KLK6 capture, finding a protein intensity (PG.Quantity) of 1117.01 in (a), and a protein intensity of 510.68 in (b) after adding 1ng of KLK6ab in both samples. The limit of detection for both machines (a) and (b) was 0.2ng of KLK6ab. Previous analyses also revealed that with a DDA analysis, the limit of detection was 5ng of KLK6ab.

**Conclusion:**

We optimized our method for identification of endogenous CSF autoantibodies on various mass spectrometric platforms using an internal antibody control that assures optimal performance of all experimental runs. We believe that this assay will be instrumental in identifying novel autoantibodies that may be related to AD pathogenesis.